# A Virtual Reality Serious Game for the Rehabilitation of Hand and Finger Function: Iterative Development and Suitability Study

**DOI:** 10.2196/54193

**Published:** 2024-08-27

**Authors:** Michael Bressler, Joachim Merk, Tanja Gohlke, Fares Kayali, Adrien Daigeler, Jonas Kolbenschlag, Cosima Prahm

**Affiliations:** 1 BG Klinik Tuebingen Clinic for Hand, Plastic, Reconstructive and Burn Surgery University of Tuebingen Tuebingen Germany; 2 Institute for Teacher Education University of Vienna Vienna Austria

**Keywords:** video games, virtual reality, exercise therapy, physical therapy, hand rehabilitation, finger rehabilitation

## Abstract

**Background:**

Restoring hand and finger function after a traumatic hand injury necessitates a regimen of consistent and conscientious exercise. However, motivation frequently wanes due to unchallenging repetitive tasks or discomfort, causing exercises to be performed carelessly or avoided completely. Introducing gamification to these repetitive tasks can make them more appealing to patients, ultimately increasing their motivation to exercise consistently.

**Objective:**

This study aims to iteratively develop a serious virtual reality game for hand and finger rehabilitation within an appealing and engaging digital environment, encouraging patient motivation for at least 2 weeks of continuous therapy.

**Methods:**

The development process comprised 3 distinct stages, each of which was subject to evaluation. Initially, a prototype was created to encompass the game’s core functionalities, which was assessed by 18 healthy participants and 7 patients with impaired hand function. Subsequently, version 1 of the game was developed and evaluated with 20 patients who were divided into an investigation group and a control group. On the basis of these findings, version 2 was developed and evaluated with 20 patients who were divided into an investigation group and a control group. Motivation was assessed using the Intrinsic Motivation Inventory (IMI), while the application’s quality was rated using the Mobile Application Rating Scale and the System Usability Scale. User feedback was gathered using semistructured interviews.

**Results:**

The prototype evaluation confirmed the acceptance and feasibility of the game design. Version 1 significantly increased motivation in 2 IMI subscales, *effort* (*P*<.001) and *usefulness* (*P*=.02). In version 2, a significant increase in daily performed exercises was achieved (*P*=.008) compared to version 1, with significantly higher motivation in the IMI subscale *effort* (*P*=.02). High Mobile Application Rating Scale scores were obtained for both versions 1 and 2, with version 2 scoring 86.9 on the System Usability Scale, indicating excellent acceptability. User feedback provided by the semistructured interviews was instrumental in the iterative development regarding improvements and the expansion of the playable content.

**Conclusions:**

This study presented a virtual reality serious game designed for hand and finger rehabilitation. The game was well received and provided an environment that effectively motivated the users. The iterative development process incorporated user feedback, confirming the game’s ease of use and feasibility even for patients with severely limited hand function.

## Introduction

### Background

Our hands are essential tools for managing daily life and are thus at high risk of injury. Therefore, comprehensive and successful rehabilitation to quickly restore hand function is essential for patients’ quality of life and ability to work. A key factor in successful rehabilitation is to maintain patients’ motivation to conscientiously participate in the process [1]. However, this is complicated by the fact that as part of their therapy, patients may experience pain reactions during or after exercise and must endure them. In addition, long-term repetitive activities are often monotonous and tend to be performed more and more carelessly without the supervision of an occupational therapist or physiotherapist [2]. However, to achieve the best possible outcome of the therapeutic process, it is necessary for patients to perform their exercises regularly, usually even daily [3,4].

In the last decade, serious games for health have become more popular and have shown a positive effect on the rehabilitation process [5,6]. The application of serious games for health covers a wide range of domains, such as training for behavioral change [7], cognitive exercises [8], the treatment of perceptual disorders [9], or physiotherapeutic exercising (eg, for pain [10] or multiple sclerosis [11,12]). The concept of gamification is an attempt to enrich a context, for example therapeutic exercising, with elements and principles used in game design [5,13]. The main target here is to positively influence the player’s attitude, enjoyment, and perceived usefulness toward the game [14]. Gamification can also contribute to improving personal health behavior [15].

The use of video games allows for the creation of exciting adventures for patients who are experiencing certain limitations due to age, illness, or disabilities and can significantly improve their mood [16]. The highly immersive experience that can be generated by the application of virtual reality (VR) technology promises to increase the positive effects on the rehabilitation process even further. The term *virtual reality* in the context of rehabilitation is often used to describe any type of computer-based system, regardless of the level of immersion. Strictly speaking, however, VR refers to a system in which the viewer is surrounded by a computer-generated 3D environment and can move around in this artificial world in real time, view it from different angles, and interact with it [17]. The cost of such immersive systems dropped dramatically after 2013, for example, a 90° field-of-view head-mounted display (HMD) was US $35,000 in 2013 and US $600 in 2016, thus enabling affordable VR hand therapy [18].

### Serious Games for Health Regarding Hand Rehabilitation

Input systems for the real-time capture of the patient’s hand and finger movements presented in the literature range from haptic devices, such as joysticks [19], robots that allow the fingers to be moved in a targeted manner [20], and data gloves [21,22], to wearable inertial tracking devices [23] and optical tracking systems with either externally placed cameras, such as the Nintendo Wii [24], or low-cost, camera-based tracking systems (eg, the Leap Motion controller that can be used stationary in front of a screen [25,26] or mounted onto a VR HMD). An alternative built-in-one setup is provided by the Meta (formerly Oculus) Quest 2 HMD [12,27]. Such markerless optical tracking generally enables a very simple setup and is also especially beneficial for patients with severely injured skin, burns, or allodynia [18,28].

Many VR and non-VR applications designed for arm and hand rehabilitation can be found in the literature, for example, for the purpose of grasping exercises after-stroke rehabilitation [21] or dexterity training for multiple sclerosis [12]. For practicing the hand and fingers in particular, several examples are given for rehabilitative tasks and activities to be performed in VR, such as playing a virtual piano, catching butterflies, picking flower petals, or solving puzzles [29,30]. Furthermore, assessment-like tasks can be found in VR, such as stacking cylinders into a pegboard or stacking cubes [31,32]. Most of these examples lack a concept that motivates the patient to stick with the game for a longer period but rather rely on the effects of technological novelty and already existing intrinsic motivation. Some tasks provide concepts, such as point systems, eventually combined with playtime and connected to a leaderboard. These rather competitive game elements influence mainly extrinsic motivation and have little effect on intrinsic motivation [33].

### Creating Motivational Serious Games for Health

Various types of players exist, and they differ in the degree to which they can be motivated by intrinsic or extrinsic motivation [34]. Although both are vital for engagement, games often focus on extrinsic motivators, such as rewards, achievements, or points, which can be harmful to intrinsic motivation [35]. Intrinsic motivation, by contrast, can be supported by self-initiation and choice [36]. Concepts from self-determination theory, such as competence, relatedness, and autonomy, can help create designs that provide sustaining engagement [37]. Game-based approaches related to self-determination theory can also be drawn from behavior change technology [38,39]. Other key factors in intrinsic motivation are informational feedback and clear game goals, which serve as proof of effectiveness for the patient and can also contribute to extending health beneficial behavior beyond the context of the game [40,41]. Work on gamification discusses the roles of intrinsic and extrinsic motivation, as both are important and not sufficiently studied empirically [33,42].

### Objectives

The primary objective of this study was to iteratively design and evaluate a serious game for the rehabilitation of hand and finger function in a patient-centered approach. In contrast to hand rehabilitation games presented in the literature, StableHandVR (BG Klinik Tübingen) had a stronger focus on different motivational factors to promote sustained user engagement for a variety of player types. Similarly, the game was designed to be feasible even for patients with severely limited hand function. The secondary objective was to compare the motivational effects of the rehabilitation game across the design iterations and a control group.

## Methods

### Ethical Considerations

Participant recruitment for the study was conducted in compliance with the Declaration of Helsinki and followed the ethical guidelines by the University of Tübingen, Germany. This study was also approved by the ethics committee of the University Clinic of Tübingen (470/2019B02). Before the initiation of the study, informed consent was obtained from all participants. All data used for this study was anonymized. No compensation was provided to the participants.

### Study Design

**Figure 1:**
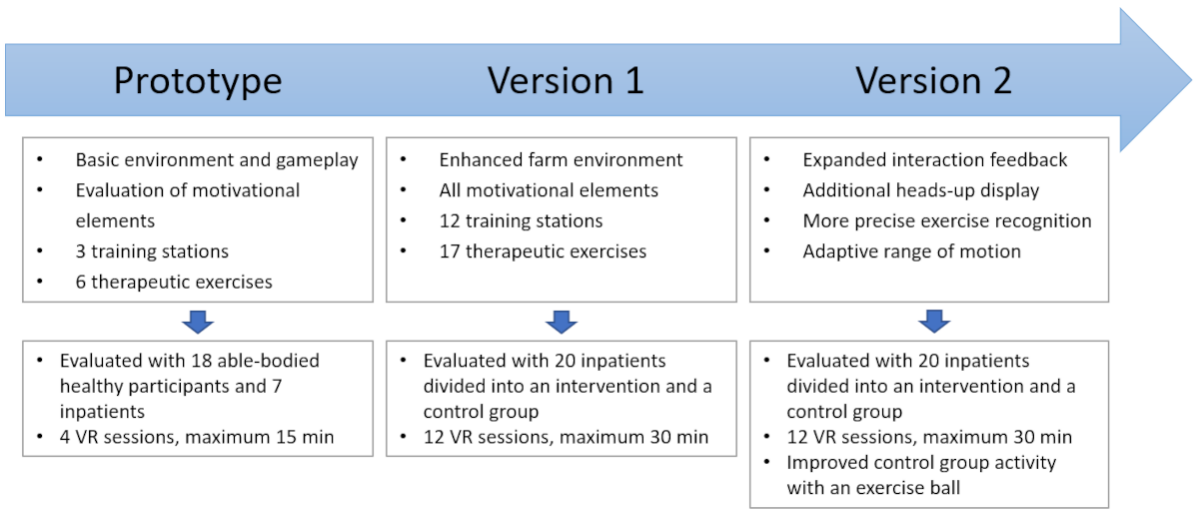
StableHandVR underwent 3 successive iterations of development and evaluation. VR: virtual reality.

This study presents the iterative design and development process of the serious game StableHandVR in 3 steps. First, a prototype was created, which provided the core game mechanics and was tested for usability and feasibility. In total, 4 game elements to maintain motivation were designed and evaluated; additional user feedback was collected. On the basis of this preliminary investigation, version 1 of the game was developed expanding the playable content to 3 weeks of training. An intervention group played the game, while a control group watched 360° videos in VR for 12 days during inpatient rehabilitation to evaluate motivational effects. Subsequently, based on the repeatedly gathered feedback and user observation, version 2 was developed and evaluated with the control group’s activity being expanded to the use of a training ball to exercise the injured hand while watching the VR videos. Figure 1 presents an overview of the 3 development stages and their evaluation.

### Apparatus and Setup

All versions of the game were developed in Unity (version 2021, Unity Technologies) for the Meta Quest 2 HMD, running as stand-alone application. The game relied primarily on the use of the inbuilt optical finger and hand tracking feature of the Meta Quest 2; no controllers were required. A physiotherapist could optionally supervise by streaming the visual contents of the HMD onto an Android tablet. The study was conducted in treatment rooms at the hospital with an exercise area of approximately 2×3 meters.

### Basic Game Design

StableHandVR aimed to transfer traditional physiotherapy hand and finger exercises into an immersive and motivating virtual world. The inspiration for placing StableHandVR in a natural environment was derived from a study conducted in the 1980s on patients undergoing postoperative recovery [43]. An early feasibility study coined the setting of the game to be a farm environment that provided several training stations to perform exercises [27]. Each station of StableHandVR included a specific task (eg, feeding and milking the cows, preparing a meal, or repairing a tractor), and its completion was divided into 6 exercises, each to be repeated 10 times. Therefore, 60 exercise repetitions had to be performed at each station, and the station’s environment would adapt with each repetition, according to the task at hand.

### Therapeutic Exercises

**Figure 2:**
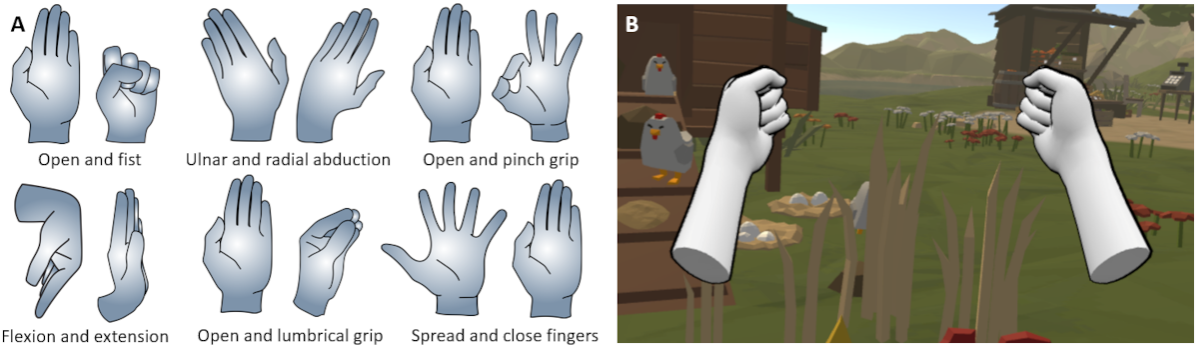
(A) Overview of the 6 exercises that were used in the prototype. (B) Preview hands as seen in version 2 demonstrated each exercise to the user.

The exercises integrated into the serious game were selected by a peer group of physiotherapists and are based on conventional hand mobility therapy [4]. They involved hand and finger movements, wrist movements, and forearm rotations. The prototype included a set of 6 basic exercises, such as closing the hand into a fist or gripping for holding a book (Figure 2).

In version 1, the number of exercises was expanded to a total of 17 different movements (a complete list is presented in Multimedia Appendix 1). Throughout the game, the difficulty of the exercises progressively increased. This was achieved by incorporating compound movements, such as simultaneously closing the hand into a fist while pronating the wrist. Moreover, each movement provided the option to be performed with both hands moving in synchrony or in opposite directions, thus adding a further level of complexity and skill requirement to the gameplay.

Performing the exercises neither involved direct interaction with the environment, such as plucking flower petals with the fingertips [44], nor a direct transfer of the patient’s hand movements to control the environment [45]. Instead, the environment would adapt automatically at each successful repetition of the exercise, according to the respective task of the station. This design decision was made to ensure good optical tracking of the hands by always being positioned to be clearly visible to the cameras. Furthermore, the original exercise should not be falsified or complicated by being combined with a virtual interaction. Moreover, this design allowed a dynamic composition of exercises for each station on each training day, adjustable for every patient.

In the prototype, preview hands were introduced that would appear in front of the player to demonstrate and clarify the requested hand movements at the beginning of each exercise. The preview hands disappeared after 2 complete repetitions. In version 2, the 3D hand model was expanded by a forearm to provide a more comprehensive visualization of the exercises containing a rotation of the wrist. In addition, instead of dark gray hands, the color was changed to a lighter gray, and the outlines of the hands were highlighted to enhance visibility (Figure 2).

### Exercise Tracking and Dynamic Range Adjustment

For the prototype, a dedicated component was developed to define and track exercise movements. This component used the hand position model supplied by the Meta Quest software development toolkit. In detail, it made use of the flexion angles of the finger joints within the provided hierarchical bone model to store and reproduce hand positions. By using the HMD, it was then possible to record various hand positions, such as an open hand or a closed fist, and subsequently use these stored positions to define exercise movements by specifying a respective start and end position as well as optional middle positions.

**Figure 3:**
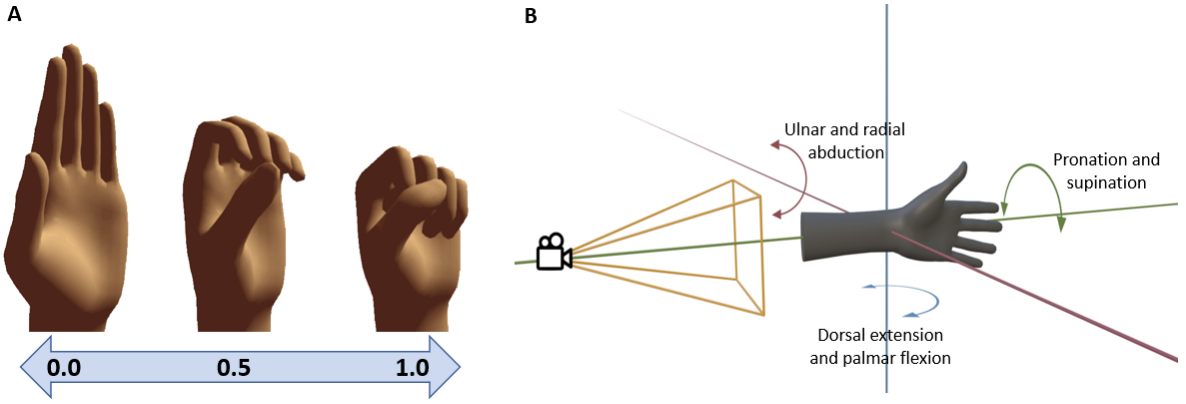
(A) For each exercise movement, a start and end position were defined to determine the position of the hands within the interval (0,1) during exercising. In the second version, additionally a middle position was defined for more accurate measurement. (B) As Meta Quest 2 does not provide wrist tracking, its rotation had to be determined using a reference coordinate system. This coordinate system had its origin at the player’s wrist and was spanned by the vertical axis (blue) and the leveled view direction (green). The third axis (red) was defined as perpendicular to the plane spanned by the first 2. This required the forearms to be held approximately along the view direction while exercising.

Due to the absence of a forearm in the tracking model of the Quest software development toolkit, the direct extraction of wrist rotation angles was not available. To compensate for this limitation, a reference coordinate system was used in replacement of a forearm bone to determine the rotation of the wrist. This coordinate system had its origin at the player’s wrist and was spanned by the vertical axis of the VR environment and a forward axis based on the player’s view direction and the forward direction of the hands, leveled within the VR environment by setting its vertical component to 0 (Figure 3). To accurately measure wrist rotation, it was necessary for the player to keep their arms bent forward during the exercise.

In addition to the interface for defining hand and finger exercises, the component was also able to track their execution. Therefore, the players’ hand positions were compared to the specified exercise and expressed as a floating-point number within the interval (ie, 0,1). In this representation, 0 denoted the start position, and 1 indicated the end position. If the hand position deviated from the movement, the position was represented as –1.

For each exercise that was newly introduced, the game initially measured the range of motion (ROM) achieved by the player as represented within (0-1). This range was subsequently used to set a minimum target for the player to be exceeded when exercising. In version 2, an adaptive approach was implemented, where the target ROM was recalculated daily, based on the average ROM achieved in the preceding days. The tracking accuracy was also enhanced in version 2 by adding an additional middle position (Figure 3) to all exercise definitions, thus providing a more precise mapping of the player’s ROM and adjustment to their skill.

### Interaction Design

**Figure 4:**
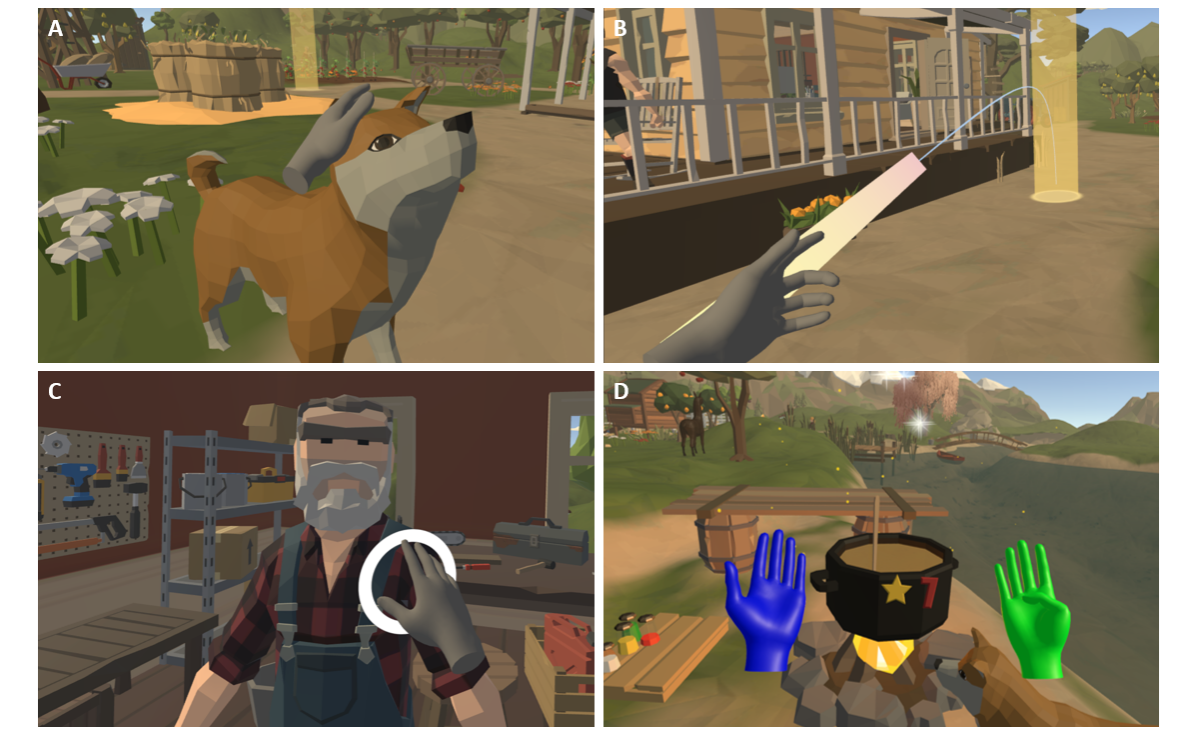
Examples of in-game interaction: (A) petting the dog, (B) teleporting, (C) interaction with a nonplayable character, and (D) performing exercises at the Fireplace station.

Outside of the exercises, the player also used their hands to interact with the game (Figure 4). In the prototype, a teleportation system was created that enabled the player to switch between stations through predefined teleport points by pointing at them. In version 1, a waypoint network was established to allow the player to explore the farm environment also beyond the stations. Starting from version 1, the player was accompanied by a dog character that would provide guidance when touched. The dog’s advice was displayed as text within a speech bubble. Nonplayable characters (NPCs) would respond to the player in a similar manner. In version 2, several improvements were implemented regarding the interaction. The teleport system was enhanced by introducing a navigation arrow with the purpose of guiding the player toward the next task. The touch interactions with the dog as well as with NPCs were improved by providing audiovisual feedback. All stations were fully supplemented with audio feedback during exercising; furthermore, an exercise counter was added that would display the number of remaining repetitions during an exercise.

### Motivational Game Elements

#### Overview

In total, 4 game elements were developed to sustain patient motivation over the course of a 3-week treatment. These different elements were designed to cater to both intrinsic and extrinsic motivation, ensuring a wide range of motivational factors. The design prioritized preventing patient frustration resulting from limited hand movement and potential therapy-related discomfort, such as pain. At the same time, the game aimed to provide a challenge to less restricted or more competitive players, ensuring they remain engaged without getting bored. Furthermore, there should be no incentive to perform the exercises sloppily, for example, a time challenge. These motivational elements underwent first evaluation in the prototype and were further refined in versions 1 and 2.

#### Storytelling

**Figure 5:**
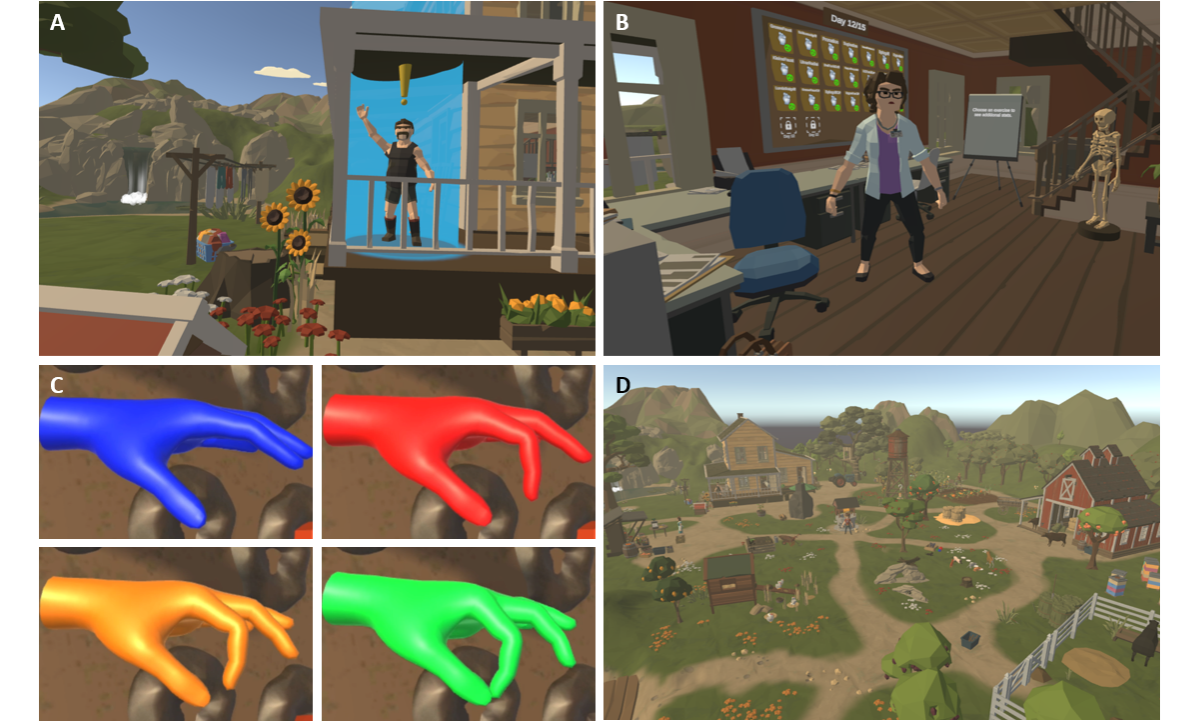
(A) A nonplayable character providing a daily task for the player. (B) The assessment station as seen in version 2 gave an overview of the player’s progress. (C) Traffic Light Hands indicate that the movements were performed well. Yellow or green color indicated that the player had reached or exceeded their personal limit. As in version 2, this limit was adjusted daily according to their previous performance. (D) From version 1, the farm contained 12 stations that were unlocked over the course of the game.

The farm was populated with NPCs, which would provide the player with daily tasks, for example, to gather carrots from the vegetable field (Figure 5). In the prototype, initially only 1 NPC was implemented. However, starting from version 1, the farm was populated with 7 NPCs who assigned the player 2 daily tasks, each involving exercises at specific stations. Once the player completed these daily tasks, they gained access to exercise at all the other unlocked stations.

#### Unlocking Rewards

Over the course of the game, the player was rewarded with additional exercise stations. In the prototype, the player unlocked a third station over the first 3 days to become playable on the fourth day. Starting from version 1, the player was rewarded with stations on each day after fulfilling their daily tasks, thus subsequently revealing all 12 stations over the course of the game (Figure 5).

#### Traffic Light Hands

Different hand colors were used to provide immediate feedback on the execution of movements. This was intended to guide the player toward performing terminal and correct movements while encouraging them to push their personal limit. A red hand color indicated that the patient reached their initially measured ROM, and the game recognized this as a successful repetition. As the patient continued to exceed this ROM, the hand color transitioned from red to orange and finally to green. In version 2, this system was adjusted daily based on the patient’s ROM (Figure 5).

#### Scoring

In the prototype, the player received score points while exercising, based on the aforementioned achieved ROM. A highscore board placed in the middle of the farm presented the scores for each exercise. In version 1, the scoring system was omitted, and in version 2, an assessment station was introduced. This station provided players with visual feedback on their ROM progress throughout the training period for each exercise (Figure 5).

### Evaluation

#### Overview

For all evaluations of StableHandVR, in-house patients from the hospital were recruited. These patients were undergoing inpatient rehabilitation because of limited hand function to such an extent that it restricted their professional and everyday activities. An overview of the type of injuries is given in Table 1. The patients took part in the VR sessions as part of their daily therapy schedule. Prerequisites for participation were basic mobility of the hand (no paralysis or total stiffness); limited hand function; no severe pain at rest (≥9 on a scale of 0-10); and the hand had to be free of stabilizing structures, such as splints, casts, or Kirschner wires. Inclusion criterion for all participants was a minimum age of 18 years.

**Table 1:** An overview of the injuries of the inpatients that were recruited for the evaluation of the prototype (n=7), version 1 (n=20), and version 2 (n=20).

Type of injury	Prototype, n (%)	Version 1, n (%)	Version 2, n (%)
Fractures in the wrist and hand area	3 (43)	11 (55)	6 (30)
Crush injuries or soft tissue injuries in the hand area	1 (14)	2 (10)	3 (15)
Tendon injuries in the area of the hand	0 (0)	2 (10)	1 (5)
Dislocations or ligament injuries in the area of the hand	1 (14)	1 (5)	1 (5)
Combination of the above points	2 (28)	4 (20)	9 (45)

#### Evaluation of the Prototype

The evaluation of the prototype involved 1 group of 25 participants consisting of 18 (72%) able-bodied individuals and 7 (28%) inpatients from the hospital (women: n=14, 56%; men: n=11, 25%. The age of the participants ranged from 18 to 56 years, with a mean age of 30.68 (SD 13.3) years. Among the 25 participants, 9 (36%) had prior experience with VR.

Each participant underwent 4 VR sessions conducted over 4 consecutive weekdays, with each session limited to 15 minutes. During each session, participants completed 1 exercise station and had the option to voluntarily complete a second one. In addition, only 1 of the 4 possible motivational game elements (Storytelling, Unlocking Rewards, Traffic Light Hands, and Scoring) was active during each of the sessions. In total, 3 motivational elements were evaluated in a randomized order over the first 3 sessions. In the fourth session, the player was consistently rewarded with access to a third exercise station, thus representing the fourth element, Unlocking Rewards.

After each session, participants rated their experience using 3 scales from the Intrinsic Motivation Inventory (IMI) questionnaire [46], specifically *interest and enjoyment,* *effort* and *pressure* on a 7-point Likert scale. In addition, participants were interviewed to gather their feedback on the game, suggestions for improvements, and ideas for additional content. The open-ended answers were evaluated based on the grounded theory [47] and thematic analysis [48].

#### Evaluation of Version 1

In total, 20 inpatients (women: n=6, 30%; men: n=14, 70%) from the hospital were equally assigned to either intervention or control group. The age ranged from 24 to 70 years, with a mean age of 48.8 (SD 12.3) years. Of the 20 inpatients, 8 (40%) had prior experience with VR.

During their 3-week inpatient rehabilitation program, both groups completed 12 VR sessions on consecutive weekdays in addition to their regular rehabilitation therapy. Each VR session was limited to 30 minutes and was supervised by a physiotherapist. In the intervention group, patients played the VR game and completed 2 mandatory tasks in each session. Additional training stations that were already unlocked could be voluntarily explored. New exercises were introduced every fourth day. In the control group, patients used the VR headset to watch a 360° video during each session, with durations ranging from 10 to 15 minutes. Following the final session, both groups were surveyed using the *interest and enjoyment*, *effort*, *usefulness*, and *pressure* subscales of the IMI questionnaire. The intervention group also evaluated the VR game using the Mobile Application Rating Scale (MARS) questionnaire [49], with the scales *engagement*, *functionality*, *aesthetics* and *impact on knowledge and attitudes* rated on a 5-point Likert scale, and was interviewed to gather feedback on the game. For further analysis of the user behavior, the game automatically recorded all user interactions in a time log.

#### Evaluation of Version 2

In total, 20 inpatients from the hospital were equally assigned to either the intervention or control group. One patient dropped out after the second session in the intervention group and another after the third session in the control group. The intervention group dropout was due to a dislike for the game, while the control group dropout was due to a transfer to another hospital. Both dropouts were replaced by 2 additional patients. All following analyses refer to the 18 remaining original patients and the 2 replacements.

The age of the final 20 patients (women: n=10, 50%; men: n=10, 50%) ranged from 22 to 61 years, with a mean age of 38.1 (SD 12.9) years; Of the 20 inpatients, 5 (25%) had prior experience with VR. The evaluation of version 2 followed a similar approach as version 1, with 2 modifications to the test protocol. First, the activity of the control group was extended by incorporating a crumple ball exercise for patients to engage their injured hand while watching the VR content. This addition aimed to provide an unspecific exercise for the injured hand. Second, patients in the intervention group had to rate the game using the System Usability Scale (SUS) [50] on a 5-point Likert scale after the last session. This measure was introduced to gather validated feedback on the usability of the game.

### Data Analysis

All statistical analyses were performed with MATLAB for Windows (version R2021a; MathWorks) with a significance level of α=.05. To assess demographic effects, patients from the evaluations of versions 1 and 2 were consolidated and categorized into 5 age groups (<30, 30-39, 40-49, 50-59, and >60 years), into a group of women or men, and a VR-experienced versus no VR-experience group. All data gathered from the automatic tracking were tested for normal distribution using the Kolmogorov-Smirnov test. Due to the small sample sizes, nonparametric tests were used for all analyses. For multiple pair-wise comparisons, the Kruskal-Wallis test was used with the *P* level adjusted by Bonferroni correction. For single pair-wise comparisons, the Wilcoxon rank sum test was used. Demographic data, temporal data, and variables gathered from questionnaires were represented as mean (SD); the number of stations per day and the duration of exercise repetitions, gathered from the automatically tracked in-game data were represented as mean (SE).

## Results

### Prototype Results

All 25 participants completed the 4 VR sessions, and none of them reported vertigo or discomfort at any point. Over the course of the 4 sessions, all participants learned to operate the game without assistance. The mean VR playtime for each session decreased from 14.5 (SD 0.65) minutes for the first session to 10.6 (SD 0.82) minutes for the last session. All participants were able to perform the 6 exercises in a way that the game could recognize them. The mean duration needed to perform 1 repetition of an exercise decreased from the first to the fourth session from 7.1 (SD 0.35) to 6 (SD 0.38) seconds for the patients and from 5.6 (SD 0.26) to 4.7 (SD 0.24) seconds for the able-bodied participants.

The evaluation of the game elements with the IMI questionnaire resulted in high scores for all 4 elements on the 2 subscales *interest and* e*njoyment* and *effort* and low scores on the *pressure* subscale (Multimedia Appendix 2). There were neither substantial differences between the elements nor over the course of the 4 days. When asked about their most favored motivational element in the interviews, the Traffic Light Hands and Unlocking Rewards both were mentioned most frequently (8 mentions), followed by Storytelling (6 mentions) and Scoring (3 mentions), which lacked significance for many participants, as they always achieved the full ROM and therefore the maximum number of points.

The overall feedback gathered from the interviews was highly positive; the farm setting displayed in the game was widely regarded as pleasant and appealing (10 mentions). The direct feedback of the Traffic Light Hands (6 mentions), the interactive aspects of the station environments that would adapt during the execution of the exercises (5 mentions), the animals on the farm including the dog companion (4 mentions), and the general idea of gamifying a rather boring rehabilitation activity (4 mentions) were also positively mentioned. The least favored experiences were the recognition of the exercises (4 mentions), and the low number of exercise stations (4 mentions). The most mentioned suggestions for improvements were more variety in general (6 mentions), more exercise stations (4 mentions), enhanced storytelling (2 mentions), more variety in exercise movements (2 mentions), and a larger farm area to explore (2 mentions). When asked about suggestions for additional content, many suggestions were made for additional training stations related to farm work, mostly regarding animals (7 mentions) but also regarding the farm infrastructure (4 mentions), such as the farmhouse or the tractor, and activities regarding the lake (3 mentions).

### Version 1 Results

**Figure 6:**
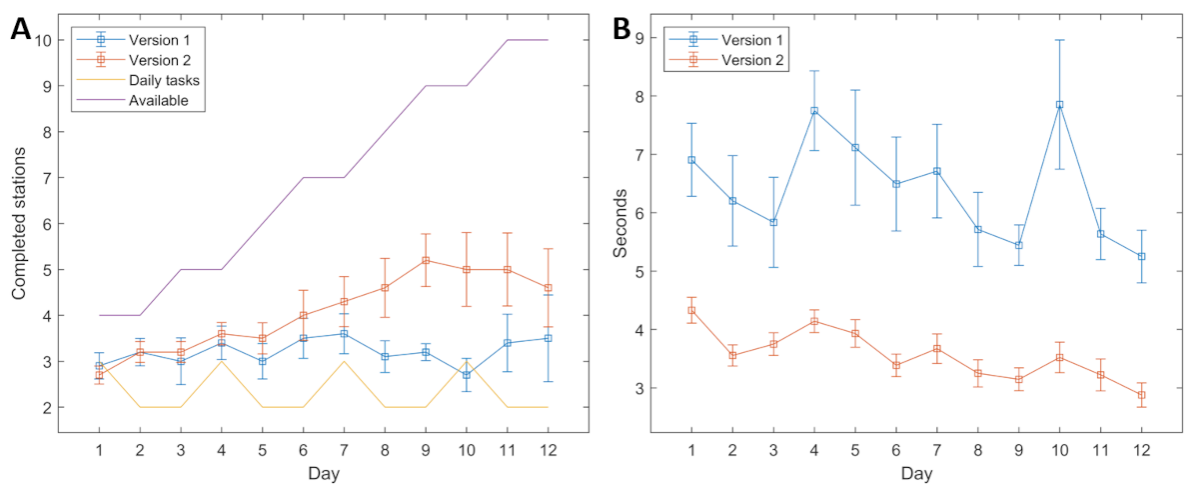
(A) The mean number and SE of completed stations in version 1 and version 2 as well as the minimum amount demanded by the daily tasks and the maximum possible number of stations for each day. (B) The mean duration and SE that were required to execute 1 repetition of 1 exercise in version 1 and version 2. New exercises were introduced on days 1, 4, 7, and 10.

All 20 patients in both groups completed the 12 VR sessions; no patient reported any experience of discomfort or motion sickness. The mean VR playtime of the intervention group was 25.3 (SD 5.9) minutes on the first day and 26.4 (SD 10.6) minutes on the last day, with a mean playtime of 22.8 (SD 7.1) minutes over all days. The mean number of stations played per day was higher than the mandatory amount specified by the daily tasks on all days except for the 1st and the 10th day (Figure 6). Due to their limited hand function, some patients had problems performing the exercises such that they were recognized by the game, especially on the 10th day when the last set of exercises was introduced. The mean duration of the execution of 1 exercise repetition increased strongly on the days when new exercises were introduced (Figure 6) but decreased over the course of the 12 days from 6.9 (SE 0.62) seconds to 5.3 (SE 0.45) seconds.

The MARS rating resulted in high scores for all subscales: *engagement* (mean 4.18, SD 0.42), *functionality* (mean 4.17, SD 0.47), *aesthetics* (mean 4.2, SD 0.48), and *impact on knowledge and attitudes* (mean 4.7, SD 0.35; Multimedia Appendix 3). All IMI subscale scores were slightly higher in the intervention group than in the control group (Multimedia Appendix 4); significant differences were found for the subscales *effort* (*P*<.001) and *usefulness* (*P*=.02).

The user observation indicated that most patients could navigate the game independently without assistance after the initial 2 days, except when new exercises were introduced. The visual and textual instructions provided by the preview hands and the dog companion were occasionally unclear, requiring clarification from the assisting physical therapist. Some patients had difficulties with orienting themselves on the farm, especially on the first days. At the training stations, it was not always clear for some patients where the action was taking place to signify task completion. Patients mentioned difficulties regarding exercise counting, and they expressed the need for a visible counter and more auditory feedback at the stations to signal when a repetition was completed.

Both groups reported perceiving the VR sessions as a vacation from their inpatient stay. They were able to momentarily forget about their injuries, felt being transported to another place, and experienced a sense of tranquility. The feedback from the intervention group regarding their game experience again was highly positive. The Traffic Light Hands were frequently mentioned for motivating patients to extend their limits toward terminal movements (6 mentions) and providing feedback on correct exercises (4 mentions); the immersive scenario, which made patients lose track of time during practice (6 mentions), and the virtual representation of their hand, which made them forget their injury (4 mentions), were also mentioned frequently. Some participants expressed their wish for an option to observe their progress, for example, as a score system that was not present in version 1.

### Version 2 Results

A total of 20 patients completed the 12 VR sessions, of whom 19 (95%) did not report any discomfort or motion sickness and 1 (5%) answered with “don’t know.” The mean VR playtime of the intervention group was 20.3 (SD 5.9) minutes on the first day and 16.7 (SD 8.5) minutes on the last day, with an overall mean of 20.9 (SD 8.9) minutes on all days. The mean number of stations played was higher than in version 1 after the second day (Figure 6), and the overall mean of stations played was significantly higher (*P*=.008). All patients were able to perform the exercises in a way that was recognized by the game. The mean duration for performing 1 exercise repetition decreased from 4.6 (SE 0.24) seconds on the first day to 2.6 (SE 0.23) seconds on the last day. On the days with new movements being introduced, the increases were not as pronounced as in version 1 (Figure 6). The overall mean duration for performing 1 exercise repetition was significantly lower than that in version 1 (*P*<.001).

Compared to version 1, the MARS rating resulted in slightly higher scores for the subscales *engagement* (mean 4.24, SD 0.71) and *functionality* (mean 4.28, SD 0.42) and in slightly lower scores for the subscales *aesthetics* (mean 4.0, SD 0.63) and *impact on knowledge and attitudes* (mean 4.14, SD 0.73; Multimedia Appendix 3). The SUS rating resulted in a mean score of 86.9 (SD 3.3), which ranges in the fourth quartile and represents excellent acceptability. The scores of the IMI subscales were higher for the intervention group (Multimedia Appendix 4) than for the control group, with significant differences for the subscale *effort* (*P*=.02).

Similar to version 1, the user observation indicated that patients were able to operate the game without assistance after the first 2 days but required some assistance in learning new exercise movements. Orientation on the farm and at the stations was comprehensible for all patients, and performing the exercises caused the patients less difficulty than in version 1. Both groups reported the relaxing effect of the VR experience, and the most mentioned categories regarding the overall feedback from the interviews were similar to version 1: the Traffic Light Hands (5 mentions), the immersive game experience (5 mentions), and the virtual representation of the hands (3 mentions). Furthermore, the execution of the exercises was mostly described as working well (5 mentions). The newly designed Scoring element, providing a progress overview of the ROM at the assessment station, experienced the same issues as in the prototype, namely, always showing the possible maximum of points for the most exercises. Therefore, it was described as not very meaningful and was predominantly not used. Suggestions were made to provide other scenarios, such as a dungeon- or sci-fi–themed environment. Patients also raised the wish for a more personalized experience, and suggestions were made, for example, custom paint for the farmhouse or customizable virtual hands. Finally, recommendations for activities regarding the lake were mentioned again, such as fishing or riding a boat.

### Demographic Effects

Regarding age, the 40- to 49-year age group showed the lowest number of completed stations among all age groups as well as the lowest IMI scores for *interest and enjoyment*, *effort*, and *usefulness*, while high scores for *pressure* were reported. The 30- to 39-year age group completed the most stations, and the <30-year age group reported the highest IMI scores for *interest and enjoyment*, *effort,* and *usefulness* and the lowest *pressure* score. Men played more stations than women patients and accordingly had higher IMI scores for *interest and enjoyment*, *effort*, and *usefulness,* with a lower *pressure* score. Patients with no prior VR experience completed more stations per day than patients with prior VR experience. However, the latter rated higher IMI scores for *interest*, *effor*t, and *usefulness* but also a higher score for the *pressure* scale. The complete list of values is presented in Table 2.

**Table 2:** Number of stations completed per day and Intrinsic Motivation Inventory scores (1-7 scale)^a^.

Demographic group		Stations per day, mean (SE)	Interest and Enjoyment, mean (SD)	Effort, mean (SD)	Pressure, mean (SD)	Usefulness, mean (SD)
**Gender**						
	Men (n=11)	3.89 (0.6)	6.4 (0.6)	6.1 (1.0)	2.0 (0.6)	6.5 (0.6)
	Women (n=9)	3.34 (0.5)	6.3 (1.1)	6.0 (1.0)	1.9 (0.7)	6.3 (1.2)
**VR^b^ Experience**						
	Prior VR experience (n=4)	3.04 (0.5)	6.4 (0.9)	6.1 (0.6)	2.2 (0.7)	6.5 (0.5)
	No prior VR experience (n=16)	3.79 (0.6)	6.3 (0.8)	6.0 (1.0)	1.9 (0.7)	6.4 (0.9)
**Age group (y)**						
	<30 (n=2)	3.58 (0.3)	6.6 (0.6)	7.0 (0)	1.5 (0.7)	6.9 (0.2)
	30-39 (n=4)	4.98 (1.5)	6.0 (0.6)	5.8 (0.7)	1.7 (0.4)	6.3 (0.6)
	40-49 (n=3)	2.97 (0.9)	5.9 (2.0)	5.7 (1.5)	2.1 (0.7)	5.5 (2.1)
	50-59 (n=8)	3.08 (0.3)	6.6 (0.5)	6.0 (1.0)	2.1 (0.8)	6.8 (0.2)
	>60 (n=3)	4.05 (1.1)	6.3 (0.3)	6.3 (0.7)	2.0 (0.3)	6.1 (0.4)

^a^The data were consolidated from version 1 (n=10) and version 2 (n=10) and categorized by demographic groups.

^b^VR: virtual reality.

## Discussion

### Principal Findings

In this study, we iteratively developed a serious health game for hand and finger rehabilitation with the deliberate goal of contributing to the long-term engagement of patients. The game was developed for the Meta Quest 2 because this HMD with built-in finger tracking allowed for a very simple and fast setup. A second reason for this decision was the finger tracking of the device, which produced better results than the Leap Motion or UltraLeap controllers when performing certain finger positions that were considered important, for example, the thumb touching ≥1 long fingers.

The iterative development and evaluation steps ensured a patient-centered design process by user observation and feedback through semistructured interviews. In general, participants reported a high level of immersion that allowed them to temporarily escape from their inpatient setting into another world. This was reported by both the intervention group and control groups and impressively confirms the potential of VR as described in the literature [18,51]. The setting of the farm environment and the display of nature were overall received very positively, a design decision that was also inspired by literature [43]. However, in the original intervention group of version 2, one patient quit after the second session because they did not like the game in general. In terms of engagement, we observed the entire spectrum from patients who tended to be underchallenged to patients well within their physical and cognitive limitations. We attribute these differences not only to varying levels of hand function but also to contrasting user preferences (eg, purpose vs mastery) [34], possibly coined by the practice of playing video games.

We expected a decrease in motivation related to the higher age of the participants and thus less familiarity with video games [52]. The familiarity was reflected by the IMI subscale *pressure*, which was clearly lowered for the <30- and 30- to 39-year age groups. However, while the 40- to 49-year and 50- to 59-year age groups completed the fewest stations per day, the >60-year age group reached the second highest value, only surpassed by the 30- to 39-year age group and before the <30-year age group. Differences in the number of stations completed between genders are smaller than between VR and no VR experience. The other IMI scales *interest*, *effort*, and *usability* show only minor deviations for all demographic groups. From these findings, it could be concluded that while age, gender, or the effect of technological novelty due to using VR for the first time might affect the overall motivation, these demographic effects did not harm the intrinsic motivation of the patients.

Compared to their control groups, higher IMI scores were measured for both intervention groups, and significant differences were found for the subscales *effort* in version 1 and version 2 and *usefulness* in version 1. It should be mentioned that, surprisingly the control groups were also very motivated to watch the videos, which again demonstrates the potential of using VR in the context of rehabilitation. However, we assume that in an unsupervised setting, the motivation in the control groups would have decreased more compared to the intervention groups [2] because the game, as used in our study, still represented a form of supervision. The assurance of performing the therapeutic exercises correctly may have been a key factor for the significantly higher IMI subscale *usefulness* of the intervention group, as scored in version 1 [41]. According to the MARS questionnaire, awareness for conscious health behavior could be raised for the intervention groups, which is linked to perceived attitude, enjoyment, and especially usefulness [14,15].

To engage a wide range of player types, 4 motivational game elements offering different motivational factors were developed. The element Storytelling should provide tasks and a context, Unlocking Rewards should offer new incentives for discovery, the Traffic Light Hands would provide direct feedback, and the Score would provide long-term feedback [34]. Among these 4, the first 3 elements can be considered rather successful, as they scored high on the IMI questionnaire and the subscales *effort* and partially also *usability* were significantly higher than in the control groups. In particular, the Traffic Light Hands received positive feedback in the interviews. This direct biofeedback, which is a critical factor for intrinsic motivation [41,53], not only encouraged patients to perform terminal movements but also gave them confidence to exercise correctly.

In contrast, the Scoring element already proved to be unsuccessful in the prototype stage because it lacked meaningfulness in terms of showing the patient’s ROM improvement. This was mainly caused by the fact that many players were able to perform most exercises with full range from the beginning. Due to the ambiguity, the element was discontinued in version 1 to avoid negative feedback and discouragement. Due to the frequent demand to visually represent the progression of ROM, the element was reintroduced in version 2 with a different design, which did not work properly for the same reasons. Therefore, the resulting lack of competitive motivational elements might be the main reason why the game was not challenging enough for some patients [34]. Furthermore, the lack of visualization of the therapy progress deprived patients of evidence of efficacy, which is also a key motivating factor for health games [41].

The comparison of versions 1 and version 2 shows a significantly higher number of stations played per day, from which it can be concluded that the overall motivation in version 2 was higher. Besides the improved interaction feedback, also reflected by a high SUS score, the most obvious improvement was the tracking accuracy of the therapeutic exercises, which can clearly be seen by the mean time that was required for a single exercise repetition being reduced by almost half. This circumstance may be mainly responsible for the fact that more stations were played. However, this interpretation must also consider that the mean age of the patients in version 2 was 10 years lower than in version 1. In both versions, a similar amount of time was spent in the game, on average approximately 22 minutes. As there were no significant differences in the IMI scores between version 1 and version 2, we conclude that the improvements did not affect the intrinsic motivation, which was already high in version 1.

### Limitations

Patients attended the VR sessions after their daily rehabilitation routine and may have been exhausted, which could potentially affect their motivation negatively. By contrast, we can assume that by being part of their daily routine, the supervised participation in VR sessions, although voluntary, was accompanied by stronger motivation than if it had been unsupervised [2]. The large variation in the degree of injury and impairment between individual patients may have influenced the outcome of measured and observed motivation. A larger sample size would better compensate for this effect.

Due to the short evaluation time of only 12 days, it was only partially possible to measure the long-term course of motivation.

### Outlook and Future Challenges

While we acknowledge the challenges in making the game equally appealing and challenging for all patients, our approach of incorporating a mix of extrinsic and intrinsic motivational elements was generally successful. By individually adjusting the composition of the movements, the challenge level of the game could be easily increased for underachieving players. It has also become apparent that there is a great need for a working score system or at least a therapy progress indicator. To build up compliance and adherence among patients, this proof of effectiveness is a key factor and needs to be improved. Due to the missing forearm tracking and inaccuracies in detecting individual phalanges [54], especially within an unusual finger or hand position, the correct execution of the patient’s movements and the assessment of their ROM was limited. We expect this feature to improve over the next few years, enabling us to overcome these limitations. In the meantime, a point system could be implemented that is simply based on the number of exercises already completed.

In the context of the general shortage of therapists, it would be beneficial to use StableHandVR in an unsupervised setting, for example, at home for several weeks, following the inpatient stay. The evaluation of StableHandVR in an unsupervised setting for an extended period would also be an exciting next step from the perspective of this study. In such a setting, we see the biggest challenge for our game, both in terms of user engagement and in substituting the physical therapist, whose assistance is currently still required for correctly learning new exercises.

Finally, StableHandVR could easily be transferred into other domains where hand and finger exercises are required, such as in stroke rehabilitation [21] or multiple sclerosis [12]. As StableHandVR allows for the simple creation of additional therapeutic exercise movements, it could be extended within a short time for other motor exercises. Furthermore, we think that the promising use of different motivational elements as in StableHandVR would also be beneficial for a variety of other applications that use gamification in a therapeutic context to achieve user engagement.

### Conclusions

This study showcased a VR game designed for hand and finger rehabilitation exercises. The iterative development process allowed user feedback to be incorporated into further development. The game was well received, offering an engaging environment and various elements that effectively motivated the users. Despite impaired hand function, the tracking of therapeutic movements proved to be reliable in operating the game. The high SUS score confirms the ease of use of the game, even for patients with physical limitations. With ongoing technical advancements in optical finger tracking, we anticipate even greater accuracy in the future, paving the way for automated medical assessments and telerehabilitation scenarios. This creates the potential for StableHandVR to become an unsupervised yet engaging VR health game for postrehabilitation home use.

## Appendix

Multimedia Appendix 1 Overview of the therapeutic exercises.

Multimedia Appendix 2 Intrinsic Motivation Inventory (IMI) scores per game element in the prototype. During the prototype evaluation, participants rated their experience for each game element using the IMI questionnaire. Error bars indicate the SD of the mean.

Multimedia Appendix 3 The mean scores and SD for version 1 and 2 rated by the intervention groups using the Mobile Application Rating Scale questionnaire.

Multimedia Appendix 4 Mean scores and SD of the evaluated Intrinsic Motivation Inventory scales of the intervention group compared to the control group for version 1 (A) and version 2 (B). Statistically significant at *P*<.05.

## Abbreviations

HMD: head-mounted display
IMI: Intrinsic Motivation Inventory
MARS: Mobile Application Rating Scale
NPC: nonplayable character
ROM: range of motion
SUS: System Usability Scale
VR: virtual reality

## References

[1] Maclean N, Pound P, Wolfe C, Rudd A (2000). Qualitative analysis of stroke patients' motivation for rehabilitation. BMJ.

[2] Minetama M, Kawakami M, Teraguchi M, Kagotani R, Mera Y, Sumiya T, Nakagawa M, Yamamoto Y, Matsuo S, Koike Y, Sakon N, Nakatani T, Kitano T, Nakagawa Y (2019). Supervised physical therapy vs. home exercise for patients with lumbar spinal stenosis: a randomized controlled trial. Spine J.

[3] Foley N, McClure JA, Meyer M, Salter K, Bureau Y, Teasell R (2012). Inpatient rehabilitation following stroke: amount of therapy received and associations with functional recovery. Disabil Rehabil.

[4] Duncan SF, Flowers CW (2015). Therapy of the Hand and Upper Extremity.

[5] Kato PM (2010). Video games in health care: closing the gap. Rev Gen Psychol.

[6] Primack BA, Carroll MV, McNamara M, Klem ML, King B, Rich M, Chan CW, Nayak S (2012). Role of video games in improving health-related outcomes: a systematic review. Am J Prev Med.

[7] Terlouw G, Kuipers D, van't Veer J, Prins JT, Pierie JP (2021). The development of an escape room-based serious game to trigger social interaction and communication between high-functioning children with autism and their peers: iterative design approach. JMIR Serious Games.

[8] Chang CH, Yeh CH, Chang CC, Lin YC (2022). Interactive somatosensory games in rehabilitation training for older adults with mild cognitive impairment: usability study. JMIR Serious Games.

[9] Stammler B, Flammer K, Schuster T, Lambert M, Karnath H (2023). Negami: an augmented reality app for the treatment of spatial neglect after stroke. JMIR Serious Games.

[10] Beltran-Alacreu H, Navarro-Fernández G, Godia-Lledó D, Graell-Pasarón L, Ramos-González Á, Raya R, Martin-Pintado Zugasti A, Fernandez-Carnero J (2022). A serious game for performing task-oriented cervical exercises among older adult patients with chronic neck pain: development, suitability, and crossover pilot study. JMIR Serious Games.

[11] Kalron A, Frid L, Fonkatz I, Menascu S, Dolev M, Magalashvili D, Achiron A (2022). The design, development, and testing of a virtual reality device for upper limb training in people with multiple sclerosis: single-center feasibility study. JMIR Serious Games.

[12] Kamm CP, Blättler R, Kueng R, Vanbellingen T (2023). Feasibility and usability of a new home-based immersive virtual reality headset-based dexterity training in multiple sclerosis. Mult Scler Relat Disord.

[13] Cugelman B (2013). Gamification: what it is and why it matters to digital health behavior change developers. JMIR Serious Games.

[14] Baptista G, Oliveira T (2019). Gamification and serious games: a literature meta-analysis and integrative model. Comput Human Behav.

[15] Johnson D, Deterding S, Kuhn KA, Staneva A, Stoyanov S, Hides L (2016). Gamification for health and wellbeing: a systematic review of the literature. Internet Interv.

[16] Russoniello CV, O’Brien K, Parks JM (2009). The effectiveness of casual video games in improving mood and decreasing stress. J Cyber Ther Rehabil.

[17] Tieri G, Morone G, Paolucci S, Iosa M (2018). Virtual reality in cognitive and motor rehabilitation: facts, fiction and fallacies. Expert Rev Med Devices.

[18] Hoffman HG, Boe DA, Rombokas E, Khadra C, LeMay S, Meyer WJ, Patterson S, Ballesteros A, Pitt SW (2020). Virtual reality hand therapy: a new tool for nonopioid analgesia for acute procedural pain, hand rehabilitation, and VR embodiment therapy for phantom limb pain. J Hand Ther.

[19] Ganjiwale D, Pathak R, Dwivedi A, Ganjiwale J, Parekh S (2018). Occupational therapy rehabilitation of industrial setup hand injury cases for functional independence using modified joystick in interactive computer gaming in Anand, Gujarat. Natl J Physiol Pharm Pharmacol.

[20] Liu S, Meng D, Cheng L, Huang F (2018). A virtual reality based training and assessment system for hand rehabilitation. Proceedings of the 9th International Conference on Intelligent Control and Information Processing.

[21] da Silva Cameirão M, Bermúdez I Badia S, Duarte E, Verschure PF (2011). Virtual reality based rehabilitation speeds up functional recovery of the upper extremities after stroke: a randomized controlled pilot study in the acute phase of stroke using the rehabilitation gaming system. Restor Neurol Neurosci.

[22] Gabyzon ME, Engel-Yeger B, Tresser S, Springer S (2016). Using a virtual reality game to assess goal-directed hand movements in children: a pilot feasibility study. Technol Health Care.

[23] Yang X, Yeh SC, Niu J, Gong Y, Yang G (2017). Hand rehabilitation using virtual reality electromyography signals. Proceedings of the 5th International Conference on Enterprise Systems.

[24] Standen P, Threapleton K, Richardson A, Connell L, Brown D, Battersby S, Platts F, Burton A (2017). A low cost virtual reality system for home based rehabilitation of the arm following stroke: a randomised controlled feasibility trial. Clin Rehabil.

[25] Alimanova M, Borambayeva S, Kozhamzharova D, Kurmangaiyeva N, Ospanova D, Tyulepberdinova G, Gaziz G, Kassenkhan A (2017). Gamification of hand rehabilitation process using virtual reality tools: using leap motion for hand rehabilitation. Proceedings of the 1st IEEE International Conference on Robotic Computing.

[26] Charles D, Pedlow K, McDonough S, Shek K, Charles T (2014). Close range depth sensing cameras for virtual reality based hand rehabilitation. J Assist Technol.

[27] Pereira MF, Prahm C, Kolbenschlag J, Oliveira E, Rodrigues NF (2020). A virtual reality serious game for hand rehabilitation therapy. Proceedings of the 2020 IEEE 8th International Conference on Serious Games and Applications for Healt.

[28] Wu YT, Chen KH, Ban SL, Tung KY, Chen LR (2019). Evaluation of leap motion control for hand rehabilitation in burn patients: an experience in the dust explosion disaster in Formosa Fun Coast. Burns.

[29] Shin JH, Kim MY, Lee JY, Jeon YJ, Kim S, Lee S, Seo B, Choi Y (2016). Effects of virtual reality-based rehabilitation on distal upper extremity function and health-related quality of life: a single-blinded, randomized controlled trial. J Neuroeng Rehabil.

[30] Then JW, Shivdas S, Tunku Ahmad Yahaya TS, Ab Razak NI, Choo PT (2020). Gamification in rehabilitation of metacarpal fracture using cost-effective end-user device: a randomized controlled trial. J Hand Ther.

[31] Postolache O, Lourenço F, Dias Pereira JM, Girão P (2017). Serious game for physical rehabilitation: measuring the effectiveness of virtual and real training environments. Proceedings of the 2017 IEEE International Instrumentation and Measurement Technology Conference.

[32] Tang HK, Feng ZQ, Xu T, Yang XH (2017). VR system for active hand rehabilitation training. Proceedings of the 4th International Conference on Information, Cybernetics and Computational Social System.

[33] Mekler ED, Brühlmann F, Tuch AN, Opwis K (2017). Towards understanding the effects of individual gamification elements on intrinsic motivation and performance. Comput Human Behav.

[34] Tondello GF, Wehbe RR, Diamond L, Busch M, Marczewski A, Nacke LE (2016). The gamification user types hexad scale. Proceedings of the 2016 Annual Symposium on Computer-Human Interaction in Play.

[35] Deci EL, Koestner R, Ryan RM (1999). A meta-analytic review of experiments examining the effects of extrinsic rewards on intrinsic motivation. Psychol Bull.

[36] Biddiss E, Irwin J (2010). Active video games to promote physical activity in children and youth: a systematic review. Arch Pediatr Adolesc Med.

[37] Ryan RM, Deci EL (2000). Self-determination theory and the facilitation of intrinsic motivation, social development, and well-being. Am Psychol.

[38] Alahäivälä T, Oinas-Kukkonen H (2016). Understanding persuasion contexts in health gamification: a systematic analysis of gamified health behavior change support systems literature. Int J Med Inform.

[39] Cheek C, Fleming T, Lucassen MF, Bridgman H, Stasiak K, Shepherd M, Orpin P (2015). Integrating health behavior theory and design elements in serious games. JMIR Ment Health.

[40] Michie S, Ashford S, Sniehotta FF, Dombrowski SU, Bishop A, French DP (2011). A refined taxonomy of behaviour change techniques to help people change their physical activity and healthy eating behaviours: the CALO-RE taxonomy. Psychol Health.

[41] Caserman P, Hoffmann K, Müller P, Schaub M, Straßburg K, Wiemeyer J, Bruder R, Göbel S (2020). Quality criteria for serious games: serious part, game part, and balance. JMIR Serious Games.

[42] Seaborn K, Fels DI (2015). Gamification in theory and action: a survey. Int J Hum Comput Interact.

[43] Ulrich RS (1984). View through a window may influence recovery from surgery. Science.

[44] Wang Z, Wang P, Xing L, Mei L, Zhao J, Zhang T (2017). Leap Motion-based virtual reality training for improving motor functional recovery of upper limbs and neural reorganization in subacute stroke patients. Neural Regen Res.

[45] Jiang Y, Li Z, He M, Lindlbauer D, Yan Y (2023). HandAvatar: embodying non-humanoid virtual avatars through hands. Proceedings of the 2023 CHI Conference on Human Factors in Computing Systems.

[46] Reynolds L, Reynolds RA, Woods R, Baker JD (2007). Measuring intrinsic motivations. Handbook of Research on Electronic Surveys and Measurements.

[47] Adams A, Lunt P, Cairns P, Cairns P, Cox AL (2008). A qualitative approach to HCI research. Research Methods for Human-Computer Interaction.

[48] Braun V, Clarke V (2006). Using thematic analysis in psychology. Qual Res Psychol.

[49] Terhorst Y, Philippi P, Sander LB, Schultchen D, Paganini S, Bardus M, Santo K, Knitza J, Machado GC, Schoeppe S, Bauereiß N, Portenhauser A, Domhardt M, Walter B, Krusche M, Baumeister H, Messner E (2020). Validation of the mobile application rating scale (MARS). PLoS One.

[50] Brooke J, Jordan PW, Thomas B, McClelland IL, Weerdmeester B (1996). SUS: a 'quick and dirty' usability scale. Usability Evaluation In Industry.

[51] Pereira MF, Prahm C, Kolbenschlag J, Oliveira E, Rodrigues NF (2020). Application of AR and VR in hand rehabilitation: a systematic review. J Biomed Inform.

[52] Koivisto J, Hamari J (2014). Demographic differences in perceived benefits from gamification. Comput Human Behav.

[53] Kayali F, Luckner N, Purgathofer P, Spiel K, Fitzpatrick G (2018). Design considerations towards long-term engagement in games for health. Proceedings of the 13th International Conference on the Foundations of Digital Games.

[54] Abdlkarim D, Di Luca M, Aves P, Maaroufi M, Yeo S, Miall RC, Holland P, Galea JM (2024). A methodological framework to assess the accuracy of virtual reality hand-tracking systems: a case study with the Meta Quest 2. Behav Res Methods.

