# Supplementary material for: A Virtual Reality Serious Game for the Rehabilitation of Hand and Finger Function: Iterative Development and Suitability Study
Source: JMIR Serious Games. 2024 Aug 27;12:e54193. doi: 10.2196/54193 (PMC11387912; doi:10.2196/54193)
Supplement: Multimedia Appendix 1 [file games_v12i1e54193_app1.pdf]

Multimedia Appendix 1: Overview of the Therapeutic Exercises

#	Position 0	Position 0.5	Position 1	Description
1	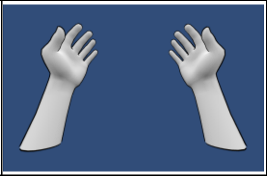	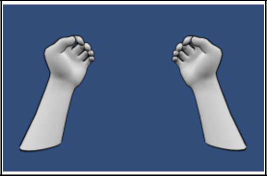	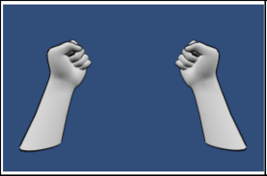	Close the hand to a fist
2	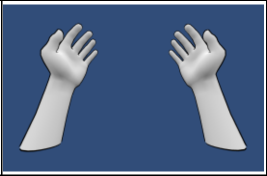	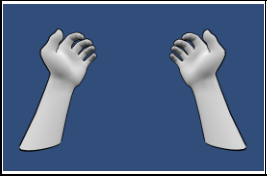	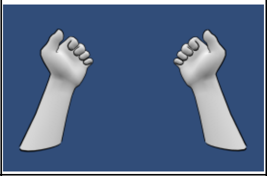	Close the long fingers to hooks
3	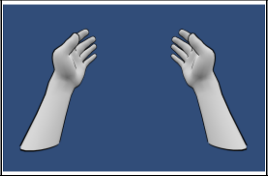	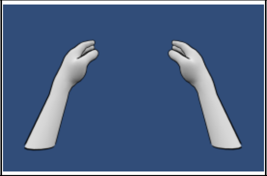	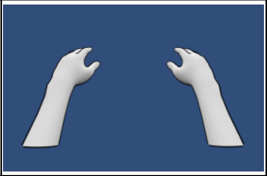	Rotate the wrist towards the thumb
4	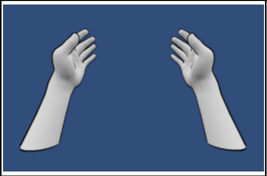	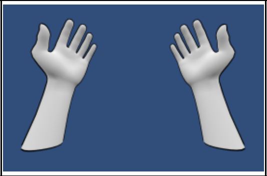	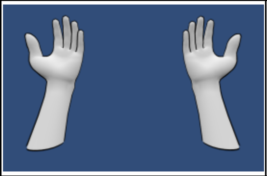	Rotate the wrist away from the thumb
5	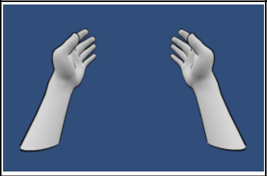	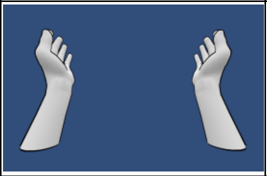	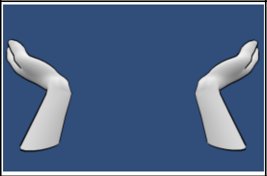	Extend the wrist towards the back of the hand
6	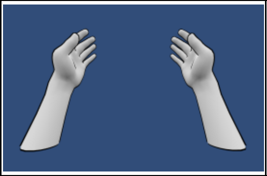	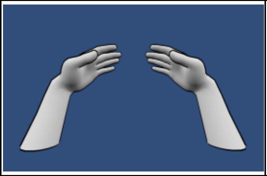	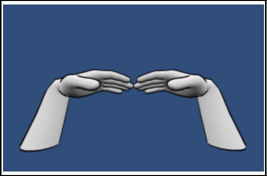	Flex the wrist towards the palm
7	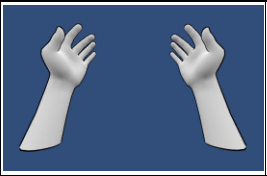	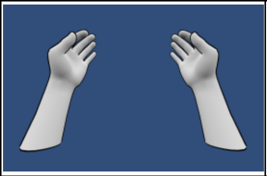	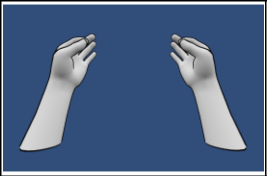	Touch the tip of the thumb and the tip of the index finger
8	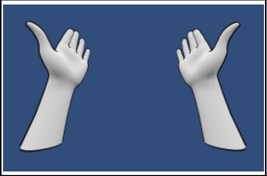	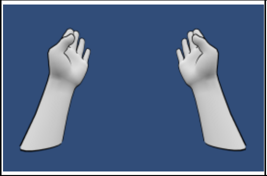	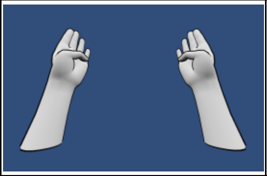	Stretch the thumb far outwards and then move it towards the little finger
9	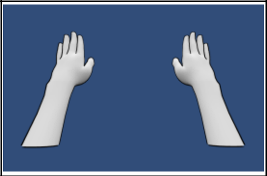	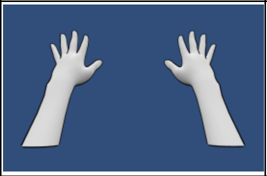	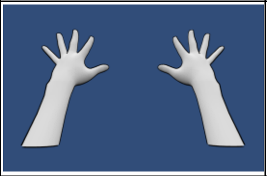	Stretch the fingers sideways
10	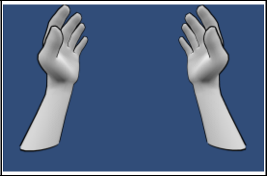	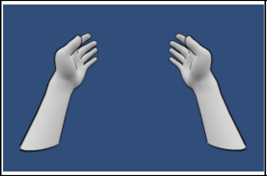	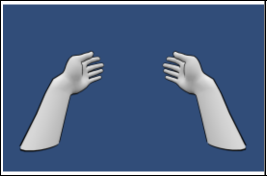	Move the wrist up and down

11	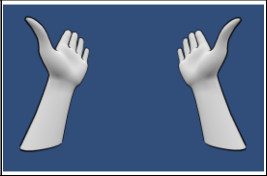	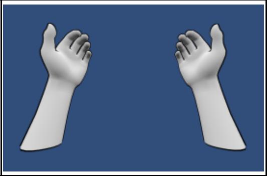	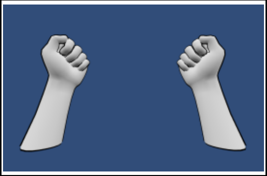	Perform a lateral grip (e.g. turning a key in the lock)
12	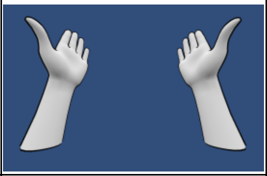	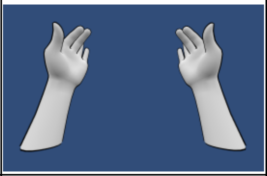	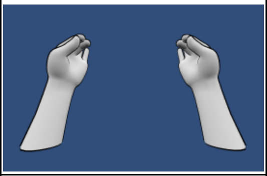	Touch the tips of thumb, index and middle finger
13	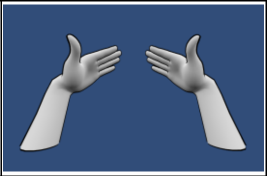	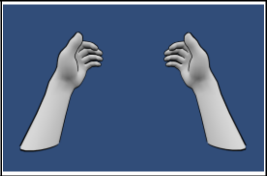	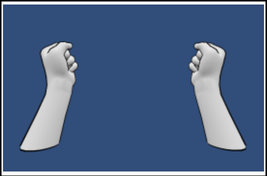	Combination of #1 (fist) and #6 (palmar flexion)
14	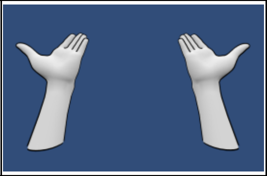	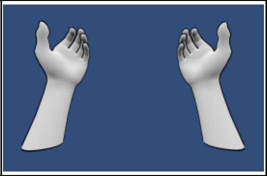	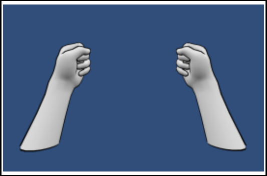	Combination of #1 (fist) and #3 (wrist pronation)
15	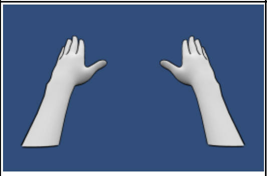	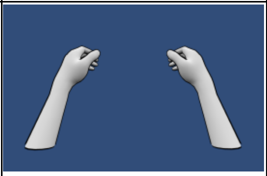	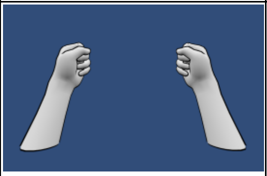	Combination of #1 (fist) and #4 (wrist supination)
16	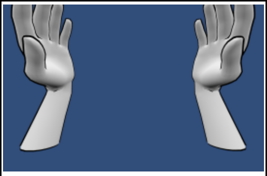	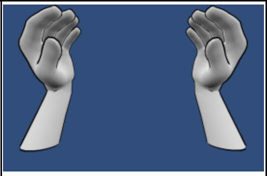	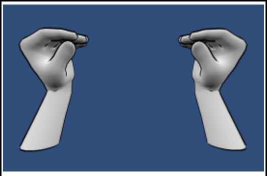	Perform a lumbrical grip, the long fingers are not bent
17	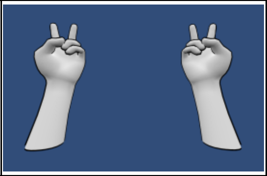	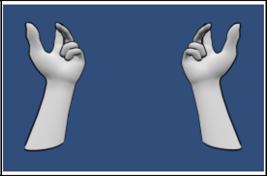	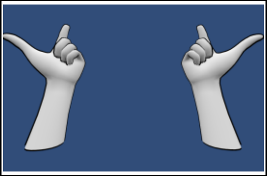	Alternate between forming a V and a L
