# Supplementary figures and images for: A Virtual Reality Serious Game for the Rehabilitation of Hand and Finger Function: Iterative Development and Suitability Study
Source: JMIR Serious Games. 2024 Aug 27;12:e54193. doi: 10.2196/54193 (PMC11387912; doi:10.2196/54193)

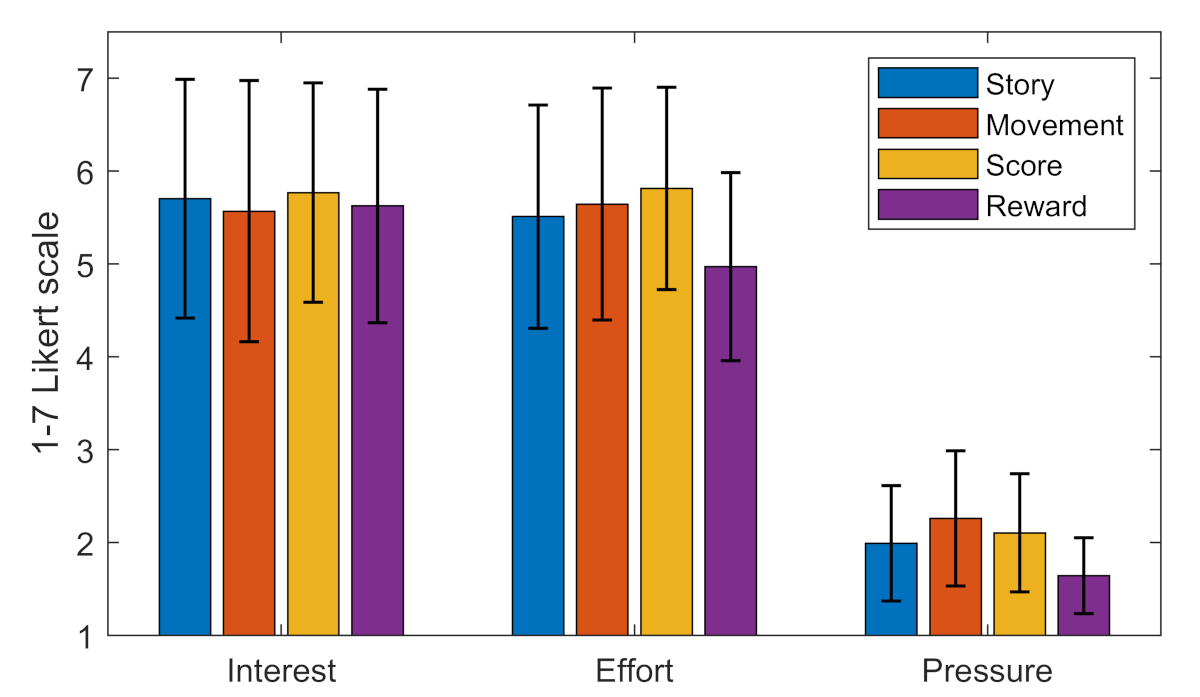

Supplement: Multimedia Appendix 2 [file games_v12i1e54193_app2.png]

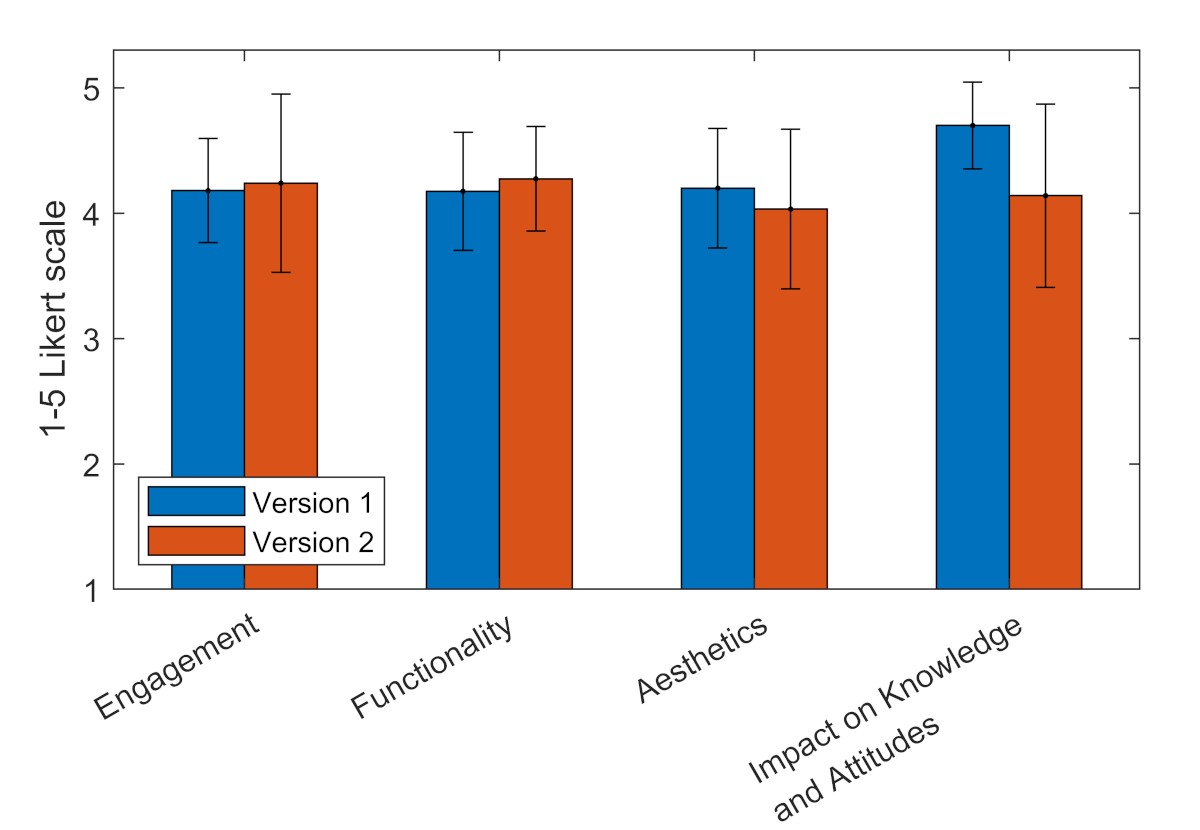

Supplement: Multimedia Appendix 3 [file games_v12i1e54193_app3.png]

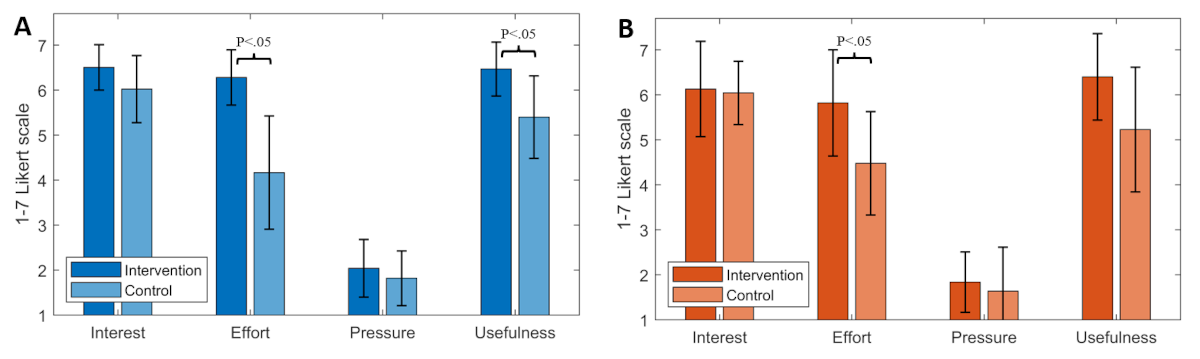

Supplement: Multimedia Appendix 4 [file games_v12i1e54193_app4.png]
